# Rising star editorial: Periacetabular osteotomy—Reliable outcome and expanding indications

**DOI:** 10.1002/jeo2.70499

**Published:** 2025-10-30

**Authors:** Lorenz Pichler, Sufian Ahmad

**Affiliations:** ^1^ Department of Orthopedics and Trauma‐Surgery Medical University of Vienna Vienna Austria; ^2^ Center for Musculoskeletal Surgery Charité ‐ Universitätsmedizin Berlin Berlin Germany; ^3^ Department of Orthopaedic Surgery Hannover Medical School Hannover Germany

**Keywords:** hip dysplasia, hip preservation, periacetabular osteotomy

## Abstract

Periacetabular osteotomy has evolved from a procedure limited to developmental dysplasia of the hip into a versatile joint‐preserving surgery for a wide range of acetabular pathomorphologies. Large‐scale studies have demonstrated its effectiveness in improving pain, function and quality of life, with long‐term survival rates approaching 68% at 20 years, though modern refinements likely yield even better results. Apart from a precise technical execution, its success depends on patient selection: while patients over 40 years old were once considered contraindications, recent evidence shows that outcomes are more closely tied to the absence of preexisting arthritis than to age. Indications have expanded to acetabular retroversion and borderline dysplasia and advances in imaging and technique, such as limited soft‐tissue dissection and refined pubic cuts, have improved safety and recovery. Furthermore, technical innovations like augmented reality promise to further enhance surgical precision in the future.

**Level of Evidence:** Level V.

**Abbreviations**
CT: computed tomography
DDH: developmental dysplasia of the hip
PAO: periacetabular osteotomy
PPAHS: post‐PAO anterior hip syndrome
PROMs: patient‐reported outcome measures
THA: total hip arthroplasty

## PERIACETABULAR OSTEOTOMY

**Figure 1:**
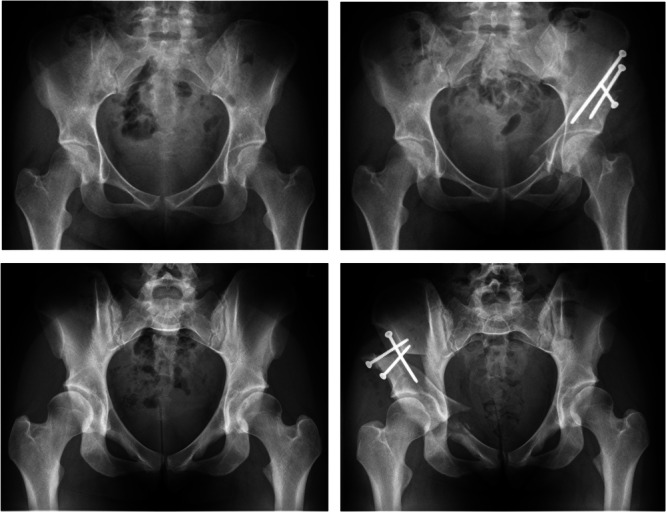
Two young female patients with symptomatic developmental dysplasia of the hip of the left (top row) and right (bottom row) hip before (left) and after (right) periacetabular osteotomy.

First described by Ganz et al., periacetabular osteotomy (PAO) has become a cornerstone joint‐preserving procedure for the treatment of symptomatic developmental dysplasia of the hip (DDH) [15]. By allowing multiplanar reorientation of the acetabulum while preserving posterior column integrity, PAO improves femoral head coverage and restores joint biomechanics (Figure 1). Over time, and supported by growing evidence in DDH, its indications have expanded to include acetabular retroversion, borderline dysplasia and complex deformities such as Perthes disease [3]. Alongside these expanded indications, patient selection criteria and surgical techniques have evolved [7, 19, 29, 37]. The result is a robust body of evidence supporting PAO as an effective and reliable procedure in a variety of indications.

## RELIABLE OUTCOME

Multiple large‐scale studies confirm the effectiveness of PAO in treating DDH. The ANCHOR study group reported on 391 hips with a mean follow‐up of 2 years, demonstrating significant short‐term improvements in pain, function and quality of life; conversion to total hip arthroplasty (THA) occurred in only 0.8% of cases, with reoperation in 3% [8]. The improvement in quality of life after PAO was further proven by studies reporting a full recovery of preoperative hip range of motion, a high return to work rate, and a significant enhancement of sexual function in these patients [14, 22, 31]. Additionally, long‐term survival is supported by a meta‐analysis of 2268 patients showing a 68% survival rate at 20 years, with conversion to THA as the endpoint [1]. However, these rates are based on cases treated in the early days of the procedure, prior to the refinement of indications and technical considerations. The survival rate of a PAO performed today is likely to be higher. If conversion to THA is eventually required, prior PAO has been associated with longer operative times and increased blood loss but no difference in clinical outcomes compared to primary THA [21].

However, as with all orthopaedic procedures, patient‐reported outcomes (PROMs) depend not only on surgical correction but also on psychosocial context. Fischer et al. found that overall mental health, pain coping strategies and baseline self‐efficacy significantly influenced postoperative satisfaction in patients undergoing PAO for DDH or retroversion [12].

## PATIENT SELECTION

Such findings underscore the importance of careful patient selection. Historically, age >40 years was considered a relative contraindication to PAO due to concerns about arthritis progression and reduced outcomes. Recent data challenge this assumption. Leopold et al. reported no statistically significant differences in outcomes between patients <30, 30–40 and >40 years [23]. Similarly, Muffly et al. found comparable rates of achieving the patient‐acceptable symptom state in patients aged 40–49 undergoing either PAO or THA [26, 37]. The absence of preexisting degenerative change is the primary determinant of successful outcomes after PAO, outweighing chronological age. However, because the prevalence of degenerative joint disease increases with advancing age, indications for PAO are correspondingly narrower in middle‑aged and older patients. Moreover, postoperative recovery after PAO depends on osseous healing and restoration of periarticular musculature, which are processes that decline with age and may therefore prolong or complicate rehabilitation. This should be considered in patient selection and preoperative counselling.

## EVOLUTION OF INDICATIONS

Initially reserved for classic DDH, PAO indications have expanded based on positive outcomes. One such extension is the treatment of acetabular retroversion, where anterior overcoverage and posterior undercoverage lead to femoroacetabular impingement and instability. Large cohort data support PAO as a valuable corrective procedure in these patients, showing survivorship comparable to traditional dysplasia cases [32, 33].

Borderline dysplasia, with a lateral centre‐edge angle typically between 18° and 25°, represents another new indication [27]. Traditionally treated with arthroscopy, borderline dysplasia (lateral centre‐edge angle 18°–25°) is now an accepted indication for PAO. A recent multicenter propensity‐matched analysis found slightly superior 5‐year PROMs for PAO compared to arthroscopy, supported by a meta‐analysis of 2075 patients [5, 28]. However, long‐term controlled studies are necessary to determine the optimal treatment for these hips.

Furthermore, advances in the understanding of hip pathomorphology in recent years have led to the phenotyping of acetabular dysplasia. It is now common to distinguish dysplastic phenotypes such as anterior undercoverage, true retroversion with deficient posterior coverage, anterolateral or posterolateral deficiency, and global dysplasia. This refined classification has enabled PAO to become a mainstay for treating a wide spectrum of acetabular morphologies [6, 17].

## REFINED SURGICAL TECHNIQUES

The expansion of its indications and advanced imaging has further refined surgical techniques for PAO. Several recent studies have investigated and proposed innovative surgical approaches and cutting techniques [9, 10, 20]. Contemporary approaches favour limited soft‑tissue dissection. Most surgeons no longer detach the rectus femoris, and modern techniques often preserve the sartorius partially or entirely, which may enhance postoperative recovery [19, 30, 34]. In addition, the use of intraoperative fluoroscopy can help expedite and optimise the execution of bony cuts. Nonetheless, PAO remains a complex procedure with a steep learning curve, requiring a relatively high case volume to limit surgical time and achieve consistently satisfactory outcomes [16].

Furthermore, post‐PAO anterior hip syndrome (PPAHS) is being increasingly recognised as a common phenomenon after PAO, peaking 3–6 months after surgery. Several mechanisms have been proposed: increased tension on the anterior soft‐tissue sling containing the iliopsoas tendon, elevation of the pubic root further tightening this structure, and callus formation during healing roughening the psoas valley and causing irritation. In addition, delayed union due to pubic bone displacement has also been suggested as a potential contributor [2, 4, 25].

In response, attention has been directed towards redefining the optimal position of the pubic cut in PAO to maximise bony contact, thereby promoting healing and remodelling [2, 4]. This refinement was largely driven by the need to address the frequent occurrence of PPAHS. CT‐based analyses identified that a lateral cut within 5 mm of the pubic root provides the most favourable conditions for osseous contact and stability [2, 36].

Looking ahead, major advances are expected from the integration of augmented reality (AR) into PAO surgery. Given the inherent complexity of the procedure—requiring the surgeon to rely on multiple senses, including visual‐spatial orientation, tactile and auditory feedback, and mental 3D reconstruction for blind cuts—AR holds promise in enhancing these capabilities. Such technologies could reduce technical challenges and improve the precision of this demanding procedure.

## COMBINATIONAL PROCEDURE

DDH frequently coexists with intra‐articular pathology, particularly labral tears and cam deformities, and PAO in combination with hip arthroscopy is increasingly common [11]. A systematic review on 355 patients proved that such combinational procedures yield satisfactory PROMs and a high return to sports rate [35]. Although there is a lack of evidence in favour of routine labrum repair alongside PAO, PAO in combination with femoral osteochondroplasty achieves positive outcomes without compromising joint preservation [13, 18, 24].

## CONCLUSION

PAO has evolved into an important joint‐preserving procedure for a broad spectrum of indications. It can be considered the working horse for the surgical correction of the variety of pathomorphological phenotypes on the acetabular side. With careful patient selection, its efficacy and reliability in DDH, acetabular retroversion and borderline dysplasia are supported by robust long‐term evidence. Advances in surgical technique and the integration of intra‐articular procedures further reinforce its role as a versatile tool in modern hip surgery.

## AUTHOR CONTRIBUTIONS

Each named author has substantially contributed to conducting the underlying research and drafting this manuscript.

## CONFLICT OF INTEREST STATEMENT

The authors declare no conflicts of interest.

## ETHICS STATEMENT

The authors have nothing to report.

## Data Availability

The authors have nothing to report.
